# Draft Genome Sequence of an Alphaproteobacterium, *Caenispirillum salinarum* AK4^T^, Isolated from a Solar Saltern

**DOI:** 10.1128/genomeA.00199-12

**Published:** 2013-02-14

**Authors:** Indu Khatri, Aditya Singh, Suresh Korpole, Anil Kumar Pinnaka, Srikrishna Subramanian

**Affiliations:** CSIR-Institute of Microbial Technology, Sector 39A, Chandigarh, India

## Abstract

We report the 4.9-Mb genome sequence of *Caenispirillum salinarum* AK4^T^ isolated from a sediment sample collected from a solar saltern at Kakinada, Andhra Pradesh, India.

## GENOME ANNOUNCEMENT

*Caenispirillum salinarum* AK4^T^, a member of the family *Rhodospirillaceae*, is a Gram-negative staining, vibrio-shaped, motile bacterium isolated from a solar saltern lake (1). The enzymes produced by this bacterium may be stable at high salinity, which may have some biotechnological relevance. The whole-genome sequencing will give us insight into the genome structure and function of the bacterium.

For this reason, we sequenced the genome of *Caenispirillum salinarum* AK4^T^ using the Illumina-HiSeq 1000 technology. Sequencing resulted in 23,935,580 high-quality paired-end reads (insert size of 350 bp) of length 101 bp. A total of 23,637,834 high-quality reads with approximately 480× coverage were assembled with CLCbio wb5 (word size 45 and bubble size 60) and generated 61 contigs (N_50_, 238,820 bp). The functional annotation was carried out by RAST (Rapid Annotation using Subsystem Technology) (2). tRNA was predicted by tRNAscan-SE (3) and rRNA genes by RNAmmer (4). The genome contains 3 rRNA genes (5S-23S-16S) and 54 aminoacyl-tRNA synthetase genes. The draft genome of *Caenispirillum salinarum* AK4^T^ consisted of 61 contigs of 4,952,365 bp with an average GC content of 68.8 mol%.

A total of 4,603 coding regions were found in the genome, where 3,130 (68%) were functionally annotated. The number of genes transcribed from positive strands were 2,276 and from negative strands were 2,327. The genome coding density is 88% with an average gene length of 938 bp. The annotated genome has 65 genes involved in virulence, disease, and defense, including 45 genes for resistance to antibiotics and toxic compounds; 48 genes are involved in motility and chemotaxis of bacteria, of which 26 are responsible for flagellar motility.

The functional comparison of genome sequences available on the RAST server revealed the closest neighbors of *Caenispirillum salinarum* to be *Rhodospirillum rubrum* ATCC 11170 (score 533) followed by *Magnetospirillum magneticum* AMB-1 (score 520), *Rhodospirillum rubrum* (score 490), *Magnetospirillum gryphiswaldense* MSR-1 (score 472), and *Alphaproteobacterium* BAL199 (score 318).

### Nucleotide sequence accession numbers.

This Whole Genome Shotgun project has been deposited at DDBJ/EMBL/GenBank under the accession no. ANHY00000000. The version described in this paper is the first version, ANHY01000000.
